# Raising Awareness About Cervical Cancer Using Twitter: Content Analysis of the 2015 #SmearForSmear Campaign

**DOI:** 10.2196/jmir.8421

**Published:** 2017-10-16

**Authors:** Philippe Lenoir, Bilel Moulahi, Jérôme Azé, Sandra Bringay, Gregoire Mercier, François Carbonnel

**Affiliations:** ^1^ Department of General Practice Montpellier University Montpellier France; ^2^ Laboratoire d'Informatique de Robotique et de Microélectronique de Montpellier, UMR 5506 Montpellier University/Centre National de la Recherche Scientifique Montpellier France; ^3^ Application des Mathématiques, Informatique et Statistique Group Paul Valéry University Montpellier France; ^4^ Public Health Department Montpellier University Hospital Montpellier France; ^5^ Centre d'Etudes Politiques de l'Europe Latine UMR 5112 Montpellier University/Centre National de la Recherche Scientifique Montpellier France; ^6^ Centre d'Evaluation des programmes de Prévention Santé Platform Paul Valéry University Montpellier 3, Montpellier University Montpellier France; ^7^ Avicenne Multiprofessional Health Center Cabestany France; ^8^ Institut du Cancer Montpellier Montpellier France; ^9^ Epsylon EA4556 Paul Valéry University Montpellier 3, Montpellier University Montpellier France

**Keywords:** uterine cervical neoplasms, Papanicolaou test, social media, early detection of cancer, health promotion, Twitter

## Abstract

**Background:**

Cervical cancer is the second most common cancer among women under 45 years of age. To deal with the decrease of smear test coverage in the United Kingdom, a Twitter campaign called #SmearForSmear has been launched in 2015 for the European Cervical Cancer Prevention Week. Its aim was to encourage women to take a selfie showing their lipstick going over the edge and post it on Twitter with a raising awareness message promoting cervical cancer screening. The estimated audience was 500 million people. Other public health campaigns have been launched on social media such as Movember to encourage participation and self-engagement. Their result was unsatisfactory as their aim had been diluted to become mainly a social buzz.

**Objective:**

The objectives of this study were to identify the tweets delivering a raising awareness message promoting cervical cancer screening (sensitizing tweets) and to understand the characteristics of Twitter users posting about this campaign.

**Methods:**

We conducted a 3-step content analysis of the English tweets tagged #SmearForSmear posted on Twitter for the 2015 European Cervical Cancer Prevention Week. Data were collected using the Twitter application programming interface. Their extraction was based on an analysis grid generated by 2 independent researchers using a thematic analysis, validated by a strong Cohen kappa coefficient. A total of 7 themes were coded for sensitizing tweets and 14 for Twitter users’ status. Verbatims were thematically and then statistically analyzed.

**Results:**

A total of 3019 tweets were collected and 1881 were analyzed. Moreover, 69.96% of tweets had been posted by people living in the United Kingdom. A total of 57.36% of users were women, and sex was unknown in 35.99% of cases. In addition, 54.44% of the users had posted at least one selfie with smeared lipstick. Furthermore, 32.32% of tweets were sensitizing. Independent factors associated with posting sensitizing tweets were women who experienced an abnormal smear test (OR [odds ratio] 13.456, 95% CI 3.101-58.378, *P*<.001), female gender (OR 3.752, 95% CI 2.133-6.598, *P*<.001), and people who live in the United Kingdom (OR 2.097, 95% CI 1.447-3.038, *P*<.001). Nonsensitizing tweets were statistically more posted by a nonhealth or nonmedia company (OR 0.558, 95% CI 0.383-0.814, *P*<.001).

**Conclusions:**

This study demonstrates that the success of a public health campaign using a social media platform depends on its ability to get its targets involved. It also suggests the need to use social marketing to help its dissemination. The clinical impact of this Twitter campaign to increase cervical cancer screening is yet to be evaluated.

## Introduction

### Background

Cervical cancer is the second most common cancer among women under 45 years of age and leads to significant mortality [1]. Cervical cancer is caused by human papillomavirus [2]. Smear test (Papanicolaou test) detects precancerous changes and early-stage cervical cancers. Its introduction has allowed a dramatic decline of cervical cancer incidence and death rates in many countries, especially the developed countries [3]. In the United Kingdom, an organized national screening program has been established in 1988. Incidence of cervical cancer in women aged 20-79 years in the United Kingdom has almost halved from 1982 to 2006, thanks to this program. However, its incidence is now rising in 20- to 29-year-olds from 1996 onward in most regions in the United Kingdom [4]. From 1999 to 2013, the number of women who did not attend their smear test for a 5-year period has progressively increased from 16% to 22% [5]. It suggests that organized screening is not intrinsically strong enough to keep a high coverage rate.

Social media would have a great potential to improve behavior change as interactive tools, encouraging participation and self-engagement instead of a descending information [6-8]. They are also seen as an opportunity to promote adherence to cancer prevention programs and a new way to screen at-risk population based on their personalized profile [9]. Facebook, Twitter, or Instagram had, respectively, more than 1.86 billion, 317 million, and 500 million monthly active users in December 2016. For Twitter, more than 500 million tweets are traded every day [10]. These social media platforms have become a valuable source of information for health professional and clinicians to effectively discover health-related topics and behaviors [11-14].

Public health campaigns have already tried to take advantage of the ability of social media to make a campaign viral. The amyotrophic lateral sclerosis (ALS) Ice Bucket Challenge’s goal was to mediatize and raise funds for the ALS association. The campaign had involved many celebrities worldwide. On September 1, 2014, more than 17 million videos had been shared on Facebook and had been watched more than 10 billion times by more than 440 million people [15]. Thanks to this campaign, more than US $100 million had been raised by the ALS association [16]. Hundreds of thousands of people had tweeted daily about ALS, which is a much higher number of tweets than those emitted about multiple sclerosis, a disease better known to the public [17]. Movember is an annual event organized each November since 2003 whose goal is to raise awareness about and raise funds for diseases affecting men such as prostate or testicle cancer. Participants let their moustache grow and post selfies on social media platforms to raise the awareness of their contacts and show their involvement in this campaign. In Denmark, after the initiation of the 2011 Movember campaign, a significant decline in the prostate-specific antigen level at referral and an increase in the number of patients referred under suspicion of prostate cancer were observed. However, only minor differences in referral patterns and prostate cancer diagnosis were detected [18]. These campaigns may be parasitized by the buzz they sought to create and may vehicle nonhealth-related messages. A content analysis of the 2013 Movember Canada campaign on Twitter showed that it did not meet the stated campaign objective of creating conversations about men’s health and, specifically, about prostate and testicular cancers [19].

To deal with the decrease of smear coverage in the United Kingdom, a Twitter campaign called #SmearForSmear has been launched in 2015 by Jo’s Cervical Cancer Trust for the European Cervical Cancer Prevention Week. Its goal was to encourage women to take a picture of themselves (selfie) showing their lipstick going over the edge and post it on Twitter with an awareness message promoting cervical cancer screening. The estimated audience was 500 million people [20].

### Objectives

The objectives of this study were to identify the tweets delivering raising awareness messages about cervical cancer screening and to understand the characteristics of Twitter users posting about this campaign.

## Methods

We conducted a 3-step content analysis of the English tweets posted on Twitter during the 2015 European Cervical Cancer Prevention Week.

### Data Collection and Extraction

To collect the tweets, we used the Twitter application programming interface. It allows the user to conduct manual searches for keywords in tweets with specific parameters such as hashtags, language, and date range. The ones used for this research were as follows: #SmearForSmear, English language, and tweets posted between January 25, 2015 and January 31, 2015, both dates inclusive (European Cervical Cancer Prevention Week). All tweets have been manually collected and assessed. Only original tweets, rather than retweets, were analyzed. In the tweets, only the verbatims were transcribed. Hashtags and content preceded by “@” were removed if that action did not make the verbatim unintelligible. We also considered all hypertexts linked to another verbatim on another Web platform (eg, Instagram). The corresponding verbatims were transcribed only if they were informative.

### Data Analysis

A total of 3019 tweets that met the search criteria were imported into Excel for data extraction. An analysis grid had been created based on the first 200 original tweets collected and thematically analyzed by 2 independent researchers to extract the themes (topics) of tweets’ verbatims and Twitter users’ statuses. Then, this grid had been tested on 50 new tweets. No new themes had been identified, confirming that category saturation was achieved [21]. The thematic analysis methodology used consists of transforming qualitative content into a quantitative form by establishing coding categories. The number of data units that fall into each coding category was counted (such as phrases, messages, and responses). Finally, they were categorized based on similar meanings and overt or inferred communication [22,23]. Themes were not restricted to preexisting themes. They emerged through an inductive process whereby open coding of data revealed themes that moved from the specific to the general [24]. The 2 researchers, both general practitioners and trained in qualitative study, elaborated a 7-theme codebook, based on tweets, to identify if the tweets delivered raising awareness messages about cervical cancer screening: incentive to carry out the smear test, evocation of smear test importance without any precision, reminder of the smear test preventive nature, reminder of the low incidence of smear test, allusion to the mortality or morbidity of cervical cancer, reminder of the incidence of cervical cancer, and testimony of an experience related to smear test or cervical cancer. If a tweet had at least one of these awareness-raising messages, it was considered a sensitizing tweet. Reproducibility of the classification of the first 300 original tweets by the 2 independent researchers was tested and calculated with Cohen’s kappa coefficient. The agreement was strong and varied between .8842 and 1.

The following information was collected about each tweet: verbatim, posting date, retrieval date, presence of a selfie with lipstick going over the edge, picture or video referring to the campaign, user’s sex, user’s location, number of followers at the date of retrieval, and user’s status. To classify the users, we used their Twitter status. If it did not exist or was incomplete, we extracted this information from links on their Twitter profile, whenever possible. The analysis grid enlisted 14 themes regarding Twitter users’ status: health company, media company, nonhealth and nonmedia company, marketing company, fashion company, blogger or YouTuber, health professional, National Health Service (NHS), politician, woman who experienced cervical cancer or who had relatives with cervical cancer, woman with an unspecified cancer or relatives with a similar status, woman who experienced an abnormal smear test, general public, and unknown. The “unknown” status was attributed when no information to categorize the user was available. Only the “unknown,” “general public,” or “NHS” statuses were exclusive.

An initial global description of the sample has been performed, using the frequencies of the different categories for the qualitative variables. As the distribution of quantitative variables was not always Gaussian (Shapiro–Wilk test), they were expressed by their mean, standard deviation, median, minimum and maximum values, and interquartile. Comparison of means was executed through the Student test when distribution was Gaussian; otherwise, it was based on Mann–Whitney test. Comparison of qualitative variables was executed through the chi-square test for parametric tests, or Fisher exact test when the conditions for applying chi-square test were not observed. A multivariate logistic modeling process was then conducted to identify the independent factors associated with the presence of a sensitizing message in the tweets and associated with each type of sensitizing message. A “step-by-step” selection procedure of the variable was used with an input and output variable set at 0.10 and 0.05, respectively. The significance threshold was set to 5%. Statistical analysis was performed by the Department of Medical Information at Montpellier Teaching Hospital with SAS version 9.4 (SAS Institute Inc).

## Results

### Study Population

A total of 3019 tweets met the search criteria; 1138 tweets were removed (retweets or copies of tweets); and 1881 tweets were analyzed.

Moreover, 608 tweets (32.32%) were sensitizing. Each of them included from 1 to 5 raising awareness message. The mean number of raising awareness messages among original tweets was 0.54 (standard deviation [SD] 0.93; Table 1). Incentive to carry out the smear test was the most frequent raising awareness message.

Main users were people from English-speaking countries. The United Kingdom accounted for 69.96% of the posted tweets, followed by the United States (8.67%) and Australia (1.06%). Nationality was unknown in 15.20% of cases. Moreover, 57.36% of users were women, and sex was unknown in 35.99% of cases. Twitter users had a mean number of followers of 44,420.8 (SD 420,819.04). A total of 54.44% of the users had posted at least one selfie with smeared lipstick. In addition, 15.63% tweets were associated with a picture or a video referring to the #SmearForSmear campaign.

### Univariate and Multivariate Analysis

Statistically significant associations between emitting sensitizing tweets and Twitter users’ status are detailed in Table 2.

The “step-by-step” selection procedure has allowed to identify independent factors influencing the sensitizing characteristic of a tweet (Table 3).

**Table 1:** Description of tweets and Twitter users.

Variable		Total, n (%)	
**Number of raising awareness messages in a tweet**			
	0		1273 (67.68)
	1		347 (18.45)
	2		149 (7.92)
	3		83 (4.41)
	4		25 (1.33)
	5		4 (0.21)
**Sensitizing tweet**		608 (32.32)	
	Incentive to carry out the smear test		440 (23.39)
	Reminder of smear test preventive nature		217 (11.54)
	Allusion to the mortality or morbidity of cervical cancer		134 (7.12)
	Testimony of an experience related to smear test or cervical cancer		92 (4.89)
	Smear test importance		63 (3.35)
	Evidence of the number of cervical cancers		41 (2.18)
	Low incidence of smear test		27 (1.44)
**Categories of Twitter users**			
	Unknown		442 (23.5)
	Nonhealth and nonmedia company		396 (21.05)
	Health company		292 (15.52)
	Blogger or YouTuber		262 (13.93)
	Media company		240 (12.76)
	Fashion activity		240 (12.76)
	Marketing activity		220 (11.70)
	National Health Service		79 (4.2)
	General public		77 (4.09)
	Woman who experienced cervical cancer or who had relatives that had experienced cervical cancer		60 (3.19)
	Health professional		53 (2.82)
	Woman who experienced an abnormal smear test		33 (1.75)
	Politician		12 (0.64)
	Woman who experienced an unspecified cancer or had relatives with a similar status		6 (0.32)

**Table 2:** Twitter users’ known characteristics.

Characteristics		Total, n (%)	*P* value
**Characteristics linked to a higher probability of emitting sensitizing tweets**			
	United Kingdom	1316 (82.51)	<.001
	Female gender	1079 (89.62)	<.001
	National Health Service	79 (4.2)	<.001
	Woman who experienced an abnormal smear test	33 (1.75)	<.001
**Characteristics linked to a higher probability of emitting nonsensitizing tweets**			
	Nonhealth or nonmedia company	396 (21.05)	<.001
	Media	240 (12.76)	.045
	Marketing activity	220 (11.70)	<.001
	Male gender	125 (10.38)	<.001

**Table 3:** Independent factors influencing the emission of sensitizing tweets.

Message of tweet, variables		Adjusted OR (95% CI)	*P* value
**Sensitizing tweet**			
	Woman who experienced an abnormal smear test	13.456 (3.101-58.378)	<.001
	Female gender	3.752 (2.133-6.598)	<.001
	United Kingdom	2.097 (1.447-3.038)	<.001
	Nonhealth or nonmedia company^a^	0.558 (0.383-0.814)	.002
**Directly encouraging people to go for a smear test**			
	Female gender	5.967 (2.606-13.659)	<.001
	Health company	2.203 (1.042-4.656)	.04
	United Kingdom	1.997 (1.320-3.021)	.001
	Selfie	1.673 (1.228-2.280)	.001
	Nonhealth or nonmass media company^a^	0.481 (0.310-0.746)	.001
**Evocation of the importance of smear test without any precision**			
	Woman who experienced an abnormal smear test	7.365 (2.314-23.436)	<.001
	National Health Service	4.266 (1.778-10.238)	.001
	United Kingdom	2.888 (1.015-8.212)	.047
	Fashion	2.724 (1.430-5.188)	.002
	Selfie	2.158 (1.163-4.002)	.001
**Reminder of the preventive aspect of smear test**			
	Woman who experienced an abnormal smear test	4.216 (1.734-10.254)	.001
	Politician	3.545 (1.028-12.221)	.045
	Female gender	2.555 (1.156-5.646)	.002
	Marketing activity^a^	0.414 (0.211-0.812)	.001
**Evocation of the mortal or morbid aspect of cervical cancer**			
	Woman who experienced an unspecified cancer or had relatives with a similar status	6.359 (1.043-38.776)	<.001
	Woman who experienced an abnormal smear test	5.591 (2.227-14.035)	<.001
	Female gender	3.396 (1.050-10.982)	.04
	Woman who experienced cervical cancer or had relatives with a similar status	2.598 (1.228-5.495)	.001
	United Kingdom	2.268 (1.069-4.808)	.03
**Reminder of the low incidence of smear test**			
	Politician	14.754 (3.074-70.816)	<.001
**Reminder of the incidence of cervical cancer**			
	General public	2.913 (1.002-8.474)	.049
	Picture or a video linked to the #SmearForSmear campaign	2.701 (1.372-5.318)	.004
**Statement from people who experienced abnormal smear test or cervical cancer**			
	Woman who experienced an abnormal smear test	65.364 (22.709-188.140	<.001
	Woman who experienced an unspecified cancer or had relatives with a similar status	14.371 (2.335-88.417)	.004
	Woman who experienced cervical cancer or had relatives with a similar status	7.641 (3.690-15.822)	<.001

^a^Statistically significant influence on the emission of nonsensitizing tweets).

## Discussion

### Principal Findings

A total of 32.32% of the tweets of the #SmearforSmear campaign were sensitizing. This result was promising as it goes well beyond the results of the 2013 Movember campaign where only 0.85% of the posted tweets may raise awareness about men’s health risks [18]. Many factors may explain this gap. On one hand, this campaign had been created using social marketing in a holistic approach. Its objective was clear, and its title referred to its objective. Jo’s Cervical Cancer Trust posted key messages reflecting the need to adhere to the screening of cervical cancer, and these messages have been reused by the participants of this campaign to fill the content of their tweets. A slogan had also been created “Attend your smear, reduce your risk,” widely retweeted in this campaign. On the other hand, this campaign created to detect an exclusive feminine cancer was based on elements of 2 women’s social construct: lipstick and selfies [25,26]. This approach was possible because this campaign had been designed for the United Kingdom, where the cervical cancer screening is organized. Targeted women automatically received a letter explaining them what to do to get screened and where. Receiving an invitation letter is an independent sensitizing factor associated with greater likelihood of cervical cancer screening [27].

As for the Twitter users, our expectations were broadly confirmed. From a general point of view, Twitter users posting sensitizing tweets were people personally involved in cervical cancer screening: women; women concerned by a feminine cancer, either for themselves or for their relatives; people living in the United Kingdom (where this English-speaking campaign took place); the NHS as a partner of this campaign; and women who experienced an abnormal smear test. As peers, women raised awareness by insisting on the preventive aspect of smear test and directly encouraged other women to attend their smear test. Peer influence is known as an important social lever for health-related behavior change [28]. Likewise, women or their relatives who experienced a pathological state (abnormal smear test, cervical cancer, or an unspecified cancer) had the greatest potential among categories of Twitter users to post a sensitizing tweet. Hashtags, such as #SmearForSmear, tend to create communities behaving as support group [29]. Unveiling elements of private life is conducive to trust and emotional bond [30]. Fashion company was a user status that has a significant potential to post tweets about the importance of smear test without any precision. Actively participating in the campaign by posting selfies and pictures or videos linked to the #SmearForSmear campaign helped in encouraging people to attend their smear test and disseminate the importance of smear test. Women’s magazines also acted as a guidebook and reinforced women’s individual responsibility to create and maintain good health for themselves and their families [31]. As for the other user categories, the raising awareness message in their tweets was in line with expectations. Politicians broadcasted information about the low incidence of smear test and how it helped preventing cervical cancer, in relation with their use of social media to communicate with the press and the public [32]. Health companies’ raising awareness message was more direct, encouraging people to attend their smear test. The general public was cautioned about the incidence of cervical cancer. The NHS insisted on the importance of smear test without giving more information. It was probably in relation to the fact that NHS was only a partner of this campaign and that it only helped disseminating it. Finally, there was a scotoma of health professionals. This status did not emerge as a relevant category. Their participation in a health campaign on social media platforms is interesting as it has been shown that the information contained in their posts are more likely to be true compared with those of other groups [33]. This underrepresentation was probably due to the shortness of the studied campaign period.

Conversely, “nonsensitizing” tweets had a much greater probability to be sent either by users not directly concerned with cervical cancer such as men (exclusively feminine cancer) or by users who participated but only broadcasted information, without getting involved: media, marketing companies, and nonhealth and nonmedia companies. It questions their participation in this campaign. Was it about an opportunistic appropriation of a viral campaign? It is probably one of the main limitations of the virality of health campaigns on social media. Most tweets posted for the 2013 Movember campaign and the breast cancer prevention month did not spark conversations about prostate and testicular cancer nor promote any specific preventive behavior about breast cancer [19,34]. They may also be an interesting lever for social stimulation.

### Strengths and Limitations

To our knowledge, no study analyzing the content of the #SmearForSmear campaign on Twitter has been published yet. Our findings are corroborated by the content analysis of others health campaigns on Twitter. We used a content analysis method based on a double analysis of the sensitizing capacity of each tweet, in an exploratory process. We also mined Twitter to gather information about users’ characteristics and complete the tweets’ content.

This highly demanding method made us decide early to restrict our study to one week. This choice was also relevant, in our opinion, as this campaign had been created for the European Cervical Cancer Prevention Week. Compared with other Twitter campaigns, our relatively high results must question its ability to keep a high proportion of sensitizing tweets in other countries (particularly where the cervical cancer screening is not organized) and if it remains high over time.

The choice to collect the tweets based on the hashtag #SmearForSmear may have limited their number, by omitting those not using it. As for the content analysis, 2 safeguards have been used: analyzing the content of tweets to create the categories before the study and evaluating the reproducibility of the classification by 2 independent researchers with Cohen’s kappa coefficient, which was strong in this study. The shortness of Twitter posts, limited to 140 characters, may have created a loss of information as users often used hyperlinks to be exempt from this limit. We then chose to manually mine Twitter to complete the tweets’ content and gather information about users’ characteristics.

### Perspectives

The #SmearForSmear campaign has allowed to disseminate sensitizing messages about cervical cancer screening and to become viral. It was based on a well-designed campaign, on a facilitating audience, and a facilitating health system using an organized screening.

Choosing a social media platform adapted to the target is a major concern for a successful campaign. Twitter is interesting as it is well suited for appointment campaigns such as #SmearForSmear or the ALS Ice Bucket Challenge. It also is a social media platform used by young adults to keep up in real time with news [35]. But its audience is mainly men, living in urban areas. Although diverse, its percentage of users with college educations and incomes over US $50,000 is much higher than those of Facebook or Instagram. Users of Instagram are mainly female, but 72% of online American adults use Facebook, and its audience is the most engaged with 70% logging on daily [35]. Health campaigns on social media platforms should be a way to reduce social inequities in health. In the United Kingdom, the main decline in screening was about 25- to 49-year-old women and black and Asiatic ethnic minorities [5]. Targeted audience must be on the social media platform chosen and then adapt to the shift of the evolution of their audiences.

The impact of facilitators is to be studied. As previously shown, many Twitter users of this campaign did not engage in this campaign as they did not post sensitizing tweets. But they participated and helped broadcasting to their audience. Models such as Cara Delevingne also posted a selfie to support the campaign and to raise awareness among her millions of followers (8.5 million in May 2017) [36]. They may boost a campaign as influencers and a role model.

Our findings show a clear need for studies that are capable of automatically analyzing the data and extracting useful insights from the #SmearForSmear Twitter campaign. We propose the use of machine learning to tackle these challenges, and we suggest 3 perspectives for future directions. First, we plan to undertake a large-scale analysis using a collection of tweets that we are currently collecting since February 2017. This analysis will include the application of the Latent Dirichlet Allocation to extract the topics emerging from the discussions about the campaign, as well as the exploration of the linguistic style of the Twitter’ users [11,37-39]. Second, we could benefit from statistical learning techniques to predict automatically the categories of all tweets about the campaign [40]. This study may allow us to assess our findings and generalize our results. We will learn a model with the one-vs-the-rest multiclass classifier based on an annotated dataset, and we will apply it on all tweets about the campaign. We will compare the results with the manual processing and annotation done so far. Moreover, within a sufficiently large dataset, we can take advantage of machine learning models to use features that are more complex to characterize the users tweeting about the campaign. We suggest focusing on user groups including health professionals, celebrities, general public, and politicians. This will lead us to understand which group of users is prominent, so that it could influence others, making them to retweet the messages relevant to the campaign, to like and reply to tweets, or more importantly donate money. Third, we plan to investigate the temporal distribution of messages to focus on the campaign dynamics over time. We may study the temporal correlations between the reactions of twitter users and real-world events such as media coverage of the campaign. This analysis is exploratory, and it could help in identifying the factors contributing to raising the awareness. For example, a televised promotion of the campaign or a promotion published by a celebrity may stimulate a huge volume of tweets and reactions online. Beyond this, we can also analyze the geographical distribution of tweets during the campaign.

Health campaigns on social networks may raise awareness of public health issues. Becoming viral is not an end in itself. Long-term effect of social media campaigns to raise people’s awareness of health conditions is to be evaluated. The ALS Ice Bucket Challenge has proven to be disappointing as after 2 years, the level of Web-related activities about ALS has remained practically the same as it was before the campaign [41]. The campaigns’ clinical impact is also yet to be evaluated. It will be a difficult task in an hyperconnected world to be able to individualize the effect. This scientific step is important to convince stakeholders, health professionals, and general public to get involved and use Web 3.0 as a collective intelligence to drive back chronic diseases, particularly for the most fragile ones.

## Abbreviations

ALS: amyotrophic lateral sclerosis
NHS: National Health Service
OR: odds ratio
